# Hepatitis E virus as a trigger for Guillain-Barré syndrome

**DOI:** 10.1186/s12883-021-02334-1

**Published:** 2021-08-06

**Authors:** Miriam Fritz-Weltin, Estelle Frommherz, Nora Isenmann, Lisa Niedermeier, Benedikt Csernalabics, Tobias Boettler, Christoph Neumann-Haefelin, Dominique Endres, Marcus Panning, Benjamin Berger

**Affiliations:** 1 Clinic of Neurology and Neurophysiology, Medical Center – University of Freiburg, Faculty of Medicine, University of Freiburg, Breisacher Str. 64, 79106 Freiburg, Germany; 2 Department of Medicine II, Medical Center – University of Freiburg, Faculty of Medicine, University of Freiburg, Freiburg, Germany; 3 Department of Psychiatry and Psychotherapy, Medical Center – University of Freiburg, Faculty of Medicine, University of Freiburg, Freiburg, Germany; 4 Institute of Virology, Medical Center – University of Freiburg, Faculty of Medicine, University of Freiburg, Freiburg, Germany

**Keywords:** Hepatitis E virus, Guillain-Barré syndrome, Acute polyneuroradiculitis, HEV seroprevalence

## Abstract

**Background:**

Hepatitis E virus (HEV) is the most common cause of acute viral hepatitis worldwide. An association with neuralgic amyotrophy and Guillain-Barré syndrome (GBS) was previously described. Concerning GBS, studies from other countries found an acute HEV infection in 5–11% of cases. However, HEV prevalence shows considerable regional variations. Therefore, we retrospectively analyzed the frequency of HEV infections in association with GBS in a monocentric cohort in Southwestern Germany.

**Methods:**

Overall, 163 patients with GBS treated in our clinic between 2008 and 2018 of whom serum and/or cerebrospinal fluid (CSF) samples were available, were identified. Serum samples were analyzed for anti-HEV immunoglobulin (Ig)M and IgG antibodies by ELISA. Additionally, both serum and cerebrospinal fluid (CSF) samples were tested for HEV RNA by PCR if IgM was positive or patients presented within the first 7 days from GBS symptom onset. A group of 167 healthy volunteers and 96 healthy blood donors served as controls.

**Results:**

An acute HEV infection was found in two GBS patients (1.2%) with anti-HEV IgM and IgG antibodies. HEV PCR in serum and CSF was negative in these two patients as well as in all other tested cases. Seroprevalences indicated that acute infection did not differ significantly from controls (0.8%). Anti-HEV IgG seroprevalence indicating previous infection was unexpectedly high (41%) and revealed an age-dependent increase to more than 50% in patients older than 60 years.

**Conclusion:**

In this study, serological evidence of an acute HEV infection in patients with GBS was rare and not different from controls. Comparing our data with previous studies, incidence rates show considerable regional variations.

## Introduction

Guillain-Barré Syndrome (GBS) is an acute inflammatory disorder of the peripheral nervous system (PNS) caused by a cross-reactive immune response following viral or bacterial infections, so-called molecular mimicry. Established pathogens associated with GBS are *C. jejuni, M. pneumoniae, Haemophilus influenza*, Cytomegalovirus (CMV), Epstein-Barr virus (EBV), Influenza A virus and Zika virus [1]. However, whereas two thirds of patients report gastrointestinal or respiratory symptoms preceding GBS, one third of cases have no symptoms of a prior infection [1].

Hepatitis E virus (HEV) is the most common pathogen of acute viral hepatitis worldwide, and genotype 3 is endemic throughout European countries with an age-dependent anti-HEV immunoglobulin (Ig)G seroprevalence in Germany of up to 25% in individuals older than 50 years [2]. Neurological manifestations occur in up to 16% of acute HEV infections [3], and in 2–7% of acute non-traumatic neurological injury acute HEV infections were previously found [4, 5]. Among neurological disorders, highest detection rates of an acute HEV infection were previously found in neuralgic amyotrophy (10–11%), another presumably post-infectious immune-mediated condition of the PNS [5–8], and in GBS [9]. With regard to the latter, an associated acute HEV infection was found in 5–11% of cases according to four studies from the Netherlands, Belgium, Japan and Bangladesh [10–13], respectively. Similar studies on a German population have not been performed thus far.

As has been described for other viral and bacterial infections, HEV potentially triggers GBS by the aforementioned molecular mimicry. In this regard, parainfectious disorders typically develop within 6 weeks after the preceding infection [1, 14]. Since HEV infections are asymptomatic in the majority of patients, they might account for some cases of GBS without preceding symptoms of an infection [9]. In addition, in some patients direct infiltration of the nerve roots due to neurotropic properties of the virus cannot be excluded [15]. Evidence supporting this arises from two case reports that found HEV RNA in the cerebrospinal fluid (CSF) of GBS patients [16, 17]. In line with this, HEV RNA has also been detected in the CSF of patients with meningoencephalitis [18] and neuralgic amyotrophy [7]. These data indicating neurotropic properties of the virus are supported by experimental studies showing replication of HEV in neuronal cells [19] as well as infiltration of the cerebrospinal compartment and replication in neurons [20, 21]. However, whereas HEV RNA was previously detected in serum samples of 23% of patients with GBS and a serology indicating an acute HEV infection, it has not been systematically investigated in the CSF in these studies [10–13], and was negative in all eight CSF samples tested.

The purpose of this study was to investigate the seroprevalence of an acute HEV infection in GBS patients in a large tertiary care university hospital in Southwestern Germany. Additionally, by extensive testing for HEV RNA in serum and CSF samples by PCR we investigated the temporal relation between both, and evidence for a direct infiltration of the central nervous system by the virus, respectively.

## Methods

### Patients

An electronic database search was performed to retrospectively identify patients who had been treated with GBS or its subtypes (e.g. Miller-Fisher syndrome, MFS) in our clinic between 2008 and 2018. Diagnosis had been made by the treating physician at the time of the acute disorder and was retrospectively verified based on electronic records by two of the authors (MFW and BB) according to the National Institute of Neurological Disorders and Stroke (NINDS) criteria [22]. Patients were included, if diagnosis could be verified, and if therapy naïve serum and/or CSF samples were available for the retrospective analysis. All demographic, clinical, laboratory and electrophysiological data were obtained from electronic records. Electrophysiological subtypes were determined according to the Hadden criteria [23]. For comparison, 263 individuals that had already been used in a previously published study [24] served as controls. These comprised 96 randomly selected blood donors and 167 healthy volunteers. The latter had been collected not specifically for this study, but in order to generate a biobank of control samples for future research purposes. The study was approved by the local ethics committee (No. 63/19). All participants gave their written informed consent.

### HEV testing

All serum samples were retrospectively tested for anti-HEV IgM and IgG antibodies using the commercial Wantai enzyme-linked immunosorbent assay (ELISA) (Sanbio, Netherlands) according to the manufacturer’s instructions. ELISA results are presented as ratios of sample optical density (OD) divided by the cut-off OD. For both IgM and IgG antibodies OD ratios > 1.1 indicated a positive result, whereas ratios < 0.9 were classified as negative. Borderline OD ratios (0.9–1.1) were classified as positive if diagnosis was supported by positive anti-HEV IgG antibodies. HEV PCR was retrospectively performed in blood and CSF samples of patients who presented within the first 7 days from symptom onset of GBS, regardless of serological status, as well as in all anti-HEV IgM positive patients. Nucleic acid extraction from serum and CSF samples was done using the QIAamp MinElute Virus Spin Kit (Qiagen, Hilden, Germany). HEV PCR was performed using the RealStar HEV RT-PCR kit (Altona Diagnostics, Hamburg, Germany) according to the manufacturer’s instruction on an ABI 7500 SDS Real-time PCR system (Thermo Fisher, Dreieich, Germany). All samples were obtained during routine diagnostic procedures and leftover material was stored at − 80 °C.

As described previously [4], *acute* HEV infection could be either *current* or *recent*. *Current* HEV infection was defined by detection of HEV RNA in serum and/or CSF regardless of the antibody status. *Recent* HEV infection was diagnosed if HEV PCR was negative and both anti-HEV IgM and IgG antibodies were positive. The isolated detection of anti-HEV IgG antibodies was classified as *distant* HEV infection.

### Statistical analysis

Categorical data were analyzed using Fisher’s exact test, continuous variables using Mann-Whitney U test. A *P* value of < 0.05 was considered as statistically significant. Statistical analysis was performed using SPSS Statistics version 21 (IBM Corp., Armonk, NY).

## Results

A total of 163 patients were included in the study. Demographic and clinical characteristics of these patients and healthy controls are specified in Table 1.

**Table 1:** Demographic and clinical characteristics of the study population and healthy controls

	GBS (*n* = 163)	Healthy controls (*n* = 263)
**Median age in years (SD; range)**	59 (17;18–87)	28 (15;18–89)
**Sex:**		
Male	95 (58.3)	119 (45.2)
Female	68 (41.7)	144 (54.8)
**GBS Variants:**		
Classical GBS	140 (85.9)	n.a.
sensorimotor	93 (57.1)	
pure motor	22 (13.5)	
pure sensory	5 (3.1)	
paraparetic	10 (6.1)	
Pharyngeal-cervical-brachial	2 (1.2)	
Bilateral facial palsy with paraesthesias	8 (4.9)	
Miller-Fisher syndrome (MFS)	22 (13.5)	
MFS-GBS overlap	1 (0.6)	
**GBS disability score at nadir:**		
**Mean (SD)**	**3.2 ± 1.2**	n.a.
**Median**	**3.0**	
0 - Healthy	–	
1 - Minor signs or symptoms of neuropathy, capable of manual work	12 (7.4)	
2 - Able to walk without support of a stick but incapable of manual work	39 (23.9)	
3 - Able to walk with a stick, appliance or support	42 (25.8)	
4 - Confined to bed or wheelchair-bound	49 (30.1)	
5 - Requiring assisted ventilation	21 (12.9)	
6 - Dead	–	
**CSF analysis (*n*** **= 161):**		
Albuminocytological dissociation	113 (70.2)	
Slightly increased cell count (5–50/μl) and increased protein level	26 (16.2)	
Increased cell count (50–100/μl) and increased protein level	2 (1.2)	
Normal	20 (12.4)	
**Electrophysiological testing:**		
Pathological	144 (88.3)	
AIDP	31 (19.0)	
Axonal-demyelinating subtype	55 (33.7)	
Predominantly demyelinating subtype	22 (13.5)	
Predominantly axonal subtype	11 (6.8)	
AMAN	7 (4.3)	
AMSAN	9 (5.5)	
Not classifiable	9 (5.5)	
Normal	19 (11.7)	

Data are listed as numbers (%) unless indicated otherwise

*AIDP* acute inflammatory demyelinating polyradiculoneuropathy, *AMAN* acute motor axonal neuropathy, *AMSAN* acute motor sensory axonal neuropathy *CSF* cerebrospinal fluid, *GBS* Guillain-Barré Syndrome, *n.a.* not applicable, *n* number, *SD* standard deviation

In total, 74 patients (45%) presented within the first 7 days from symptom onset of GBS. In these patients, HEV PCR was performed regardless of serological status in serum (all 74 samples available) and CSF (only 73 samples available).

**Fig. 1:**
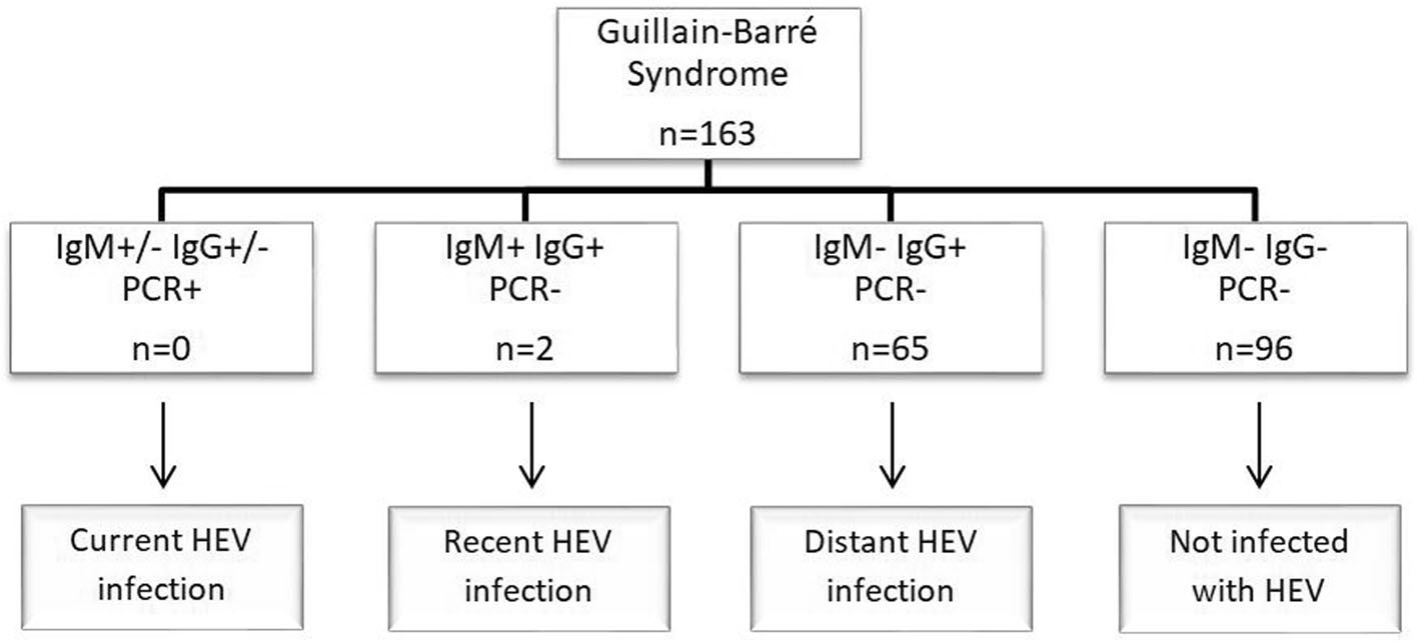
Results of Hepatitis E virus testing in the study population characterized in Table 1. *HEV* Hepatitis E Virus, *n* number, *PCR* polymerase chain reaction

Evidence of an acute HEV infection was found in two patients (1.2%; Fig. 1).

In both, detection of anti-HEV IgM and IgG antibodies but negative HEV PCR in serum and CSF led to the diagnosis of a recent HEV infection, with patient 2 showing only a borderline result of anti-HEV IgM antibodies (OD ratio 1.06). Detailed demographic and clinical characteristics of these two patients are presented in Table 2.

**Table 2:** Clinical and diagnostic features of patients with an acute HEV infection

	Patient 1	Patient 2
**Age, sex**	56, female	65, male
**Subtype**	AMSAN	Classical GBS
**Symptoms**		
Cranial Nerves	Diplopia, right-sided trigeminal hypesthesia	–
Motor	Flaccid tetraparesis, areflexia	Slight weakness in both arms, areflexia
Sensory	Hypesthesia of both legs	Hypesthesia of distal extremities, pallhypesthesia with gait ataxia
Autonomic	Reduced vital capacity	–
**GBS disability index**		
Nadir	5	2
Discharge	4	1
**Time from symptom onset to analysis**	6 weeks	5.5 weeks
**Medical History**	Type 2 diabetes, arterial hypertension, hypercholesterolemia, diabetic polyneuropathy, vitamin B12 deficiency, alcohol abuse, chronic pancreatitis, former Hepatitis A and B infection, former pontine stroke	Arterial hypertension, monoclonal gammopathy type IgG lambda
**Travel history**	None	None
**HEV Status**		
Serology		
IgM (AU/ml)	Positive (7.54)	Positive (1.06)
IgG (AU/ml)	Positive (17.78)	Positive (14.60)
PCR serum/CSF	Negative/negative	Negative/negative
**CSF**		
WBC (/μl)	3	3
Protein (mg/l)	448	600 (↑)
Q_Alb_ (×10^−3^)	7.2	7.1
**Liver function tests**		
AST (U/l)	24	37
ALT (U/l)	10	40
**Antibodies**		
(GM1,GQ1b, GD1)	Negative	Not available
**Electrophysiology**		
Demyelination	–	++
Axonal Damage	++	+

*Normal range: AST/ALT: female* 10–35 U/l, *male* 10–50 U/l, *CSF: WBC* < 5/μl*, total protein* < 450 mg/l, *Q*_*Alb*_*: 15–40 years* 6.5 × 10^− 3^, *40–59 years* 8 × 10^− 3^, *60–79 years* 9.3 × 10^− 3^

*ALT* Alanine Aminotransferase, *AST* Aspartate Aminotransferase, *CSF* cerebrospinal fluid, *HEV* Hepatitis E Virus, *PCR* polymerase chain reaction, *Q*_*Alb*_ quotient of albumin, *WBC* white blood cell count

Both revealed no clinical symptoms of acute hepatitis. HEV PCR was negative in all serum (*n* = 74) and CSF samples (*n* = 73) of patients presenting within 7 days from GBS symptom onset.

Two samples in the control group, stemming from a 31-year-old female and a 24-year-old male, respectively, revealed positive IgM and IgG antibodies (0.8%). Seroprevalence of an acute HEV infection was not significantly different from patients with GBS (*p* = 0.639). While IgM OD ratios in GBS patients were heterogeneous with one case being clearly positive (OD ratio 7.54), and the other having a borderline result (OD ratio 1.06), OD ratios of the two healthy controls were both slightly above the cut-off (OD ratio 1.55 and 1.32, respectively). IgG OD ratios were clearly positive and in the same range in all four individuals with an acute HEV infection (17.78 and 14.60 in GBS cases, 15.93 and 14.16 in controls). Due to low case numbers, statistical analysis for comparison of Ig levels was not performed.

**Fig. 2:**
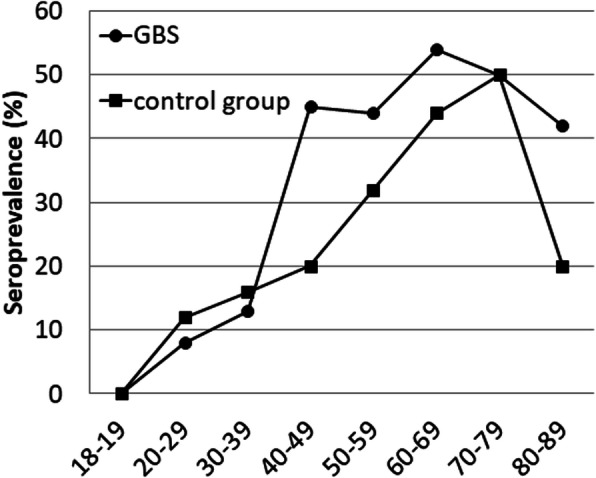
Age-adjusted IgG seroprevalence in the study population and control group characterized in Table 1

Strikingly, anti-HEV IgG was found in 67 patients (41%) and age-dependent seroprevalence showed a continuous increase with age with a peak of 54% in the seventh decade (60 to 69 years; Fig. 2, Table 3). In comparison, anti-HEV IgG antibodies were present in 17% of healthy controls only, yet these were significantly younger (Table 1). However, controls also showed an age-dependent increase of anti-HEV IgG seroprevalence up to 50% in the eighth decade (70–79 years; Fig. 2, Table 3).

**Table 3:** Age-adjusted anti-HEV-IgG seroprevalence in the study population and control group characterized in Table 1

Age group	GBS: n(%)	Healthy controls: n(%)	***P*** value^**a**^
18–19	0/3 (0)	0/2 (0)	1
20–29	1/12 (8)	18/149 (12)	1
30–39	2/15 (13)	7/45 (16)	1
40–49	9/20 (45)	4/20 (20)	.176
50–59	15/34 (44)	10/31 (32)	.445
60–69	19/35 (54)	4/9 (44)	.716
70–79	16/32 (50)	1/2 (50)	1
80–89	5/12 (42)	1/5 (20)	.600

^a^Statistical analysis: two-tailed Fisher’s exact Test

Age-dependent comparison of the two groups showed a tendency towards higher IgG prevalence in the GBS group aged 40–69 years without reaching statistical significance. IgG levels did not differ significantly between GBS patients and controls (mean ± SD: 9.7 ± 6.1 versus 9.1 ± 5.6).

## Discussion

This study investigated the prevalence of an HEV infection in patients with GBS who had been treated in our large tertiary care university hospital in Southwestern Germany. Acute HEV infection was very rare in our study population (1.2%), particularly in view of the high seroprevalence of anti-HEV IgG antibodies (41%) indicating generally high HEV infection rates in our region. Based on positive IgM antibodies, both patients in our study had recent HEV infection, whereas none had evidence of current HEV infection as would have been indicated by positive PCR. With regards to considerable geographic differences in HEV infection rates [9], this is to the best of our knowledge the first study on this issue in a German population, whereas previous studies were performed in the Netherlands, Belgium, Japan and Bangladesh [10–13].

Apart from the high number of both patients (*n* = 163) and controls (*n* = 263) a particular strength of our study was the – compared to previous studies – low threshold to perform HEV PCR in both IgM positive patients and patients with negative serology presenting within 7 days after GBS symptom onset. The latter approach was chosen in order to cover “early cases” of an infection before seroconversion could take place. These cases might be missed if diagnosis would be exclusively based on serology [25, 26], as done in previous studies [10, 11]. In this regard, our approach is in line with current guidelines recommending both serology and PCR testing [27]. For better understanding, the time course of various diagnostic markers of an HEV infection is described in more detail further below.

We regarded comparison of our results with those two studies from Europe, i.e. the Netherlands and Belgium [10, 11], appropriate, since these comply with ours concerning expected relative frequencies of GBS subtypes and HEV genotype (genotype 3 predominant in Europe) that both show relevant geographic differences [9, 23]. In addition, demographic parameters of patients were similar with a median age in the sixth decade and predominance of the male gender. Furthermore, disease severity in the Belgian cohort [11] was comparable (median GBS disability index of 3), whereas this data was not available for the Dutch study [10]. In contrast to our data, the Dutch and Belgian studies found an acute HEV infection more frequently, namely in 5% (10/201) and 8% (6/73) of GBS patients, respectively. Also in contrast to our data, in the former study by van den Berg et al. [10] acute HEV infections were more frequent in GBS patients in comparison to controls (1/201 controls, 0.5%) whereas controls were missing in the Belgian study. In addition to regional variations in infection rates, there are other possible explanations for these divergent results: (1) diagnostic criteria for HEV infection, (2) clinical characteristics of the study population, and (3) serological assays used. In view of diagnostic criteria for acute HEV infection, Stevens et al. (Belgium) based the diagnosis exclusively on positive IgM antibodies [11], whereas van den Berg et al. (Netherlands) used similar criteria as applied in our study, namely positive IgM antibodies in combination with either positive IgG or HEV PCR [10]. Our approach increases the specificity of the result, since HEV IgM antibodies show relevant cross-reactivity with other pathogens [28]. Therefore, as the authors state themselves, the Belgian study might contain false-positive cases, particularly with regards to two patients who showed positive results for other infectious triggers of GBS with known cross-reactive antibodies (CMV, EBV) [28]. Furthermore, clinical characteristics varied between study populations. Van den Berg et al. (Dutch study) included patients with classical GBS only, whereas both Stevens et al. (Belgian study) as well as our study also included patients with MFS and other rarer variants (acute motor axonal neuropathy, AMAN, and acute motor sensory axonal neuropathy, AMSAN), which constitute a minority of GBS patients [11, 12, 23]. Overall, relative frequencies of subtypes in our study were representative, since they were consistent with previous epidemiological studies from Europe [23, 25]. Finally, use of different assays for HEV antibody testing might affect results [29]. The *recom*Well assay (Mikrogen) used in the Belgian study has higher sensitivity for anti-HEV IgM, but lower sensitivity for anti-HEV IgG compared to the Wantai assay used in the Dutch and our study [29, 30]. To summarize these comparisons of serological testing, our study reveals highest concordance with the Belgian study by Stevens et al. with regards to similar demographic (age, sex) and clinical (GBS subtype and severity) characteristics of the study population. Therefore, different prevalence of an acute HEV infection (1.2% vs. 8%) might be due to regional differences, but might also in part be due to different assays, since the Belgian study used a test with higher sensitivity for IgM antibodies, which potentially increases the risk for false positive results. By contrast, the Dutch study by van den Berg et al. that also revealed a higher IgM seroprevalence (5%) used the same assay as in our study, but included patients diverging with regards to clinical characteristics (patients with classical GBS only). However, it is rather speculative, whether these clinical differences explain differences in the seroprevalence of an acute HEV infection, since none of the studies compared seroprevalence rates of patients with classical GBS and its variants.

The basic question arising from ours as well as previous studies is on how to “prove” an association between HEV infection and GBS assuming that the latter has a parainfectious etiology. For this, HEV infection would have to precede GBS by approximately three to 6 weeks, which is the typical time interval given for other infectious triggers (e.g. gastrointestinal or respiratory tract infections) [1, 14]. However, HEV infections are asymptomatic in 95% of cases [27]. Hence, determining the onset of an HEV infection and therefore temporal relation with GBS, particularly in retrospect, is impossible on clinical grounds, but relies on laboratory tests, i.e. serological and PCR testing. In this regard, incubation time of the virus is 15 to 60 days [27]. Early in the course of the infection, PCR becomes positive, and remains positive for three to 6 weeks (so-called viremia) [27, 31]. In comparison, IgM and IgG antibodies become positive in the second week of viremia, whereas they might be still negative in “very early cases” of an infection [26, 32, 33]. Interestingly, such “very early cases” during the phase of viremia with positive PCR and negative IgM antibodies have been described in HEV-associated neurological injury [4]. These cases were covered by our approach requiring PCR testing in all patients presenting during early stages, i.e. within 7 days from GBS symptom onset. Furthermore, IgM might remain positive for up to 12 months (three to 4 months on average), and IgG even for years [27, 34]. Hence, in retrospect and particularly in asymptomatic cases of an HEV infection, a positive HEV PCR would best determine a close temporal relation and therefore association to GBS, whereas in cases of positive IgM and IgG antibodies, but negative PCR, this would still be possible, but not “proven” [18, 27]. In this regard, HEV PCR indicating current infection was positive in none of our patients, 3/10 IgM positive patients in the Dutch study [10] and was not performed in any of the six IgM positive patients in the Belgian study [11]. Consequently, current HEV infection in close temporal association to GBS is not excluded in the other IgM positive patients, but has thus far only been proven in three patients from the Dutch cohort.

With regards to potential pathophysiological mechanisms, by which HEV might trigger GBS, HEV PCR was negative in all 75 CSF samples tested. Since also in former systematic studies testing for HEV RNA in GBS patients [10, 12] none had positive PCR in CSF samples indicating direct infection, the virus most likely triggers the disorder indirectly by molecular mimicry as has been described for other pathogens [1]. In contrast, evidence for direct viral infiltration of the nervous system in the clinical setting in GBS patients as would be suggested by its neurotropic properties based on experimental studies [19–21] remains thus far anecdotal [16, 17].

The reason for the unexpectedly high IgG seroprevalence in our study remains unclear, even when taking seroprevalence variability dependent on birth year, age [2] and serological assay [29] into consideration. It was comparable to the Dutch study (46%) [10], but considerably higher in comparison to the Belgian study (18%) [11], and to previous studies investigating infection rates in the general population in the Netherlands (29%) [35], in Belgium (4–14%) [36, 37], and in Germany (6–24%) [2, 38]. However, high seroprevalence rates indicate high infection rates and thus further emphasize the importance of HEV as a potential trigger of neurological disorders.

The current study has several limitations due to the retrospective, monocentric design. Specifically, serum and CSF samples were not available in some patients, and information about risk factors for HEV infection as consumption of uncooked meat was missing. Additionally, patients were not systematically screened for other infectious triggers of GBS in order to estimate the frequency of potentially cross-reactive antibodies, and the control group was not age- and sex-matched. Concerning the monocentric design, our tertiary care university hospital has a very large catchment area of approximately 2,500,000 inhabitants, and therefore we would regard it as representative for the region of Southwestern Germany.

In summary, in our cohort seroprevalence of an acute HEV infection in patients with GBS was rare and not different from controls. Furthermore, a close temporal association as would be suggested by positive HEV PCR could not be established in any of these patients. Therefore, our data apparently challenge current European guidelines, which – based on data from previous studies – generally recommend HEV testing in GBS patients [27]. Nevertheless, high IgG seroprevalence in our cohort and the control group argues for generally high infection rates with HEV in Southwestern Germany. This underlines the importance to gain more information about the association with GBS as a severe and potentially life-threatening disease that can lead to long-term disability [15]. In this regard, large prospective multicenter studies would be necessary to draw more reliable conclusions.

## Data Availability

The datasets used and analyzed during the current study are available from the corresponding author on reasonable request.
